# Supplementary material for: The Utilization and Impact of Dopamine Transporter Imaging in Diagnosing Movement Disorders at a Tertiary Care Hospital in Greece
Source: Biomedicines. 2025 Apr 16;13(4):970. doi: 10.3390/biomedicines13040970 (PMC12024717; doi:10.3390/biomedicines13040970)
Supplement: Supplementary file 1 [file biomedicines-13-00970-s001.zip › biomedicines-3542428-supplementary.pdf]

**Table S1: Results of the multiple linear regression model assessing the effect of numerous variables on the interval between symptom onset and DaTscan referral**				
**Predictor**	**β Estimate**	**Std. Error**	**95% CI**	**p-value**
Intercept	1.77	0.85	[0.975, 3.45]	0.0381
Referral Symptom: Bradykinesia	0.02	0.45	[-0.864, 0.91]	0.959
Referral Symptom: Dystonia	1.99	0.76	[0.484, 3.51]	**0.00984**
Referral Symptom: Postural Instability	0.04	0.89	[-1.71, 1.80]	0.960
Referral Symptom: Rigidity	-0.15	0.40	[-0.952, 0.634]	0.694
Male Sex	-0.26	0.26	[-0.789, 0.256]	0.317
Age	0.02	0.01	[-0.008, 0.048]	0.167
Change in Diagnosis: Yes	-1.32	0.38	[-2.08, -0.575]	**0.000592**
Pre-scan Diagnosis: Essential Tremor/Dystonia	1.49	0.55	[0.406, 2.57]	**0.00718**
Pre-scan Diagnosis: Non-Neurodegenerative Parkinsonism	-0.22	0.60	[-1.41, 0.961]	0.712
Pre-scan Diagnosis: Atypical Parkinsonism	-0.88	0.91	[-2.67, 0.911]	0.334
Pre-scan Diagnosis: Parkinson's Disease	-1.08	0.51	[-2.09, -0.079]	**0.0345**
Pre-scan Diagnosis: Vascular Parkinsonism	-1.10	1.12	[-3.30, 1.09]	0.324
Tremor as Reference in the Referral Symptom category; Unclear diagnosis as Reference in the Pre-scan Diagnosis category				

**Table S2: Results of the logistic regression model assessing the effect of numerous variables on the OR of diagnosis change following DaTscan**				
**Predictor**	**β Estimate**	**Std. Error**	**95% CI**	**p-value**
Intercept	1.34	1.05	[-0.708, 3.45]	0.201
Pre-scan Diagnosis: Essential Tremor/Dystonia	-2.90	0.61	[-4.21, -1.75]	**< 0.001**
Pre-scan Diagnosis: Non-Neurodegenerative Parkinsonism	-2.89	0.69	[-4.35, -1.59]	**< 0.001**
Pre-scan Diagnosis: Atypical Parkinsonism	-5.11	0.72	[-6.65, -3.79]	**< 0.001**
Pre-scan Diagnosis: Parkinson's Disease	-6.49	0.74	[-8.11, -5.15]	**< 0.001**
Male Sex	-0.50	0.37	[-1.24, 0.222]	0.177
Age (y)	0.02	0.02	[-0.018, 0.062]	0.297
DaTscan result: Abnormal	1.90	0.50	[0.958, 2.93]	**0.00014**
Interval between Symptom Onset and DaTscan referral	-0.32	0.15	[-0.656, -0.0675]	**0.0334**
Unclear diagnosis as Reference in the Pre-scan Diagnosis category				
